# Lagerindicine, a New Pyrrole Alkaloid Isolated from the Flowers of *Lagerstroemia indica* Linnaeus

**DOI:** 10.1007/s13659-020-00273-x

**Published:** 2020-10-20

**Authors:** Yi Chen, Song-Wei Li, Fang-Zhou Yin, Min Yang, Xia-Juan Huan, Ze-Hong Miao, Xiao-Ming Wang, Yue-Wei Guo

**Affiliations:** 1 State Key Laboratory of Drug Research, Shanghai Institute of Materia Medica, Chinese Academy of Sciences, 555 Zu Chong Zhi Road, Zhangjiang Hi-Tech Park, Shanghai, 201203 China; 2 Hunan Academy of Forestry, 658 South Shao shan Road, Changsha, 410004 Hunan China; 3 Changsha Engineering Technology Research Center of Woody Flower, 658 South shao shan Road, Changsha, 410004 Hunan China; 4 College of Materials Science and Engineering, Central South University of Forestry and Technology, 498 South Shao shan Road, Changsha, 410004 Hunan China

**Keywords:** *Lagerstroemia indica* Linnaeus, Pyrrole alkaloid, Total synthesis, Stereochemistry, Cytotoxicity

## Abstract

**Abstract:**

A phytochemical investigation of the EtOH extract of the flowers of *Lagerstroemia indica* L. led to the isolation and characterization of a new pyrrole alkaloid, named lagerindicine (**1**), along with four known compounds (**2**–**5**). Their structures were elucidated by the detailed spectroscopic analysis and comparison with literature data, whereas the structure, in particularly, the absolute configuration (AC) of **1**, was firmly determined by total synthesis. All the isolates were evaluated for their cytotoxic effects against human colon cancer cell (HCT-116), and compound **3** exhibited weak cytotoxicity with IC_50_ value of 28.4 μM.

**Graphic Abstract:**

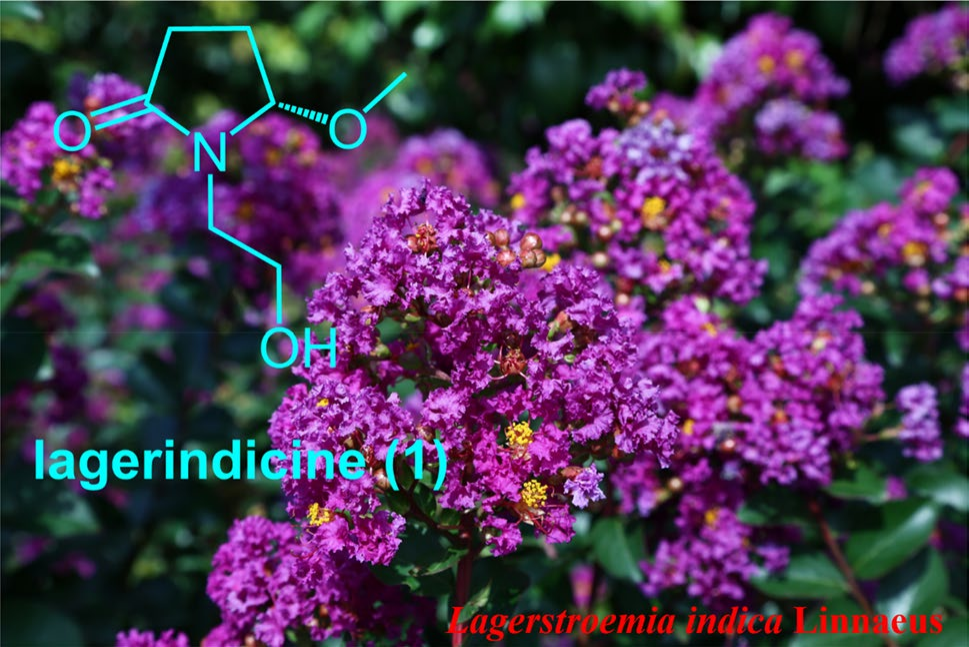

**Electronic supplementary material:**

The online version of this article (10.1007/s13659-020-00273-x) contains supplementary material, which is available to authorized users.

## Introduction

Terrestrial plants of Lythracea is a large family, widely used in architecture, landscaping, food and drug applications. The plants of genus *Lagerstroemia* comprise more than 15 species, which are either small trees or shrubs with colourful flowers distributed in warm temperate regions of China [1]. Earlier chemical investigation of the different species of Lythracea can trace back to decades, and a lot of structurally diverse and complex compounds, such as alkaloids, sesqui-, triterpenoids, steroids and flavonoids [2] were reported. Especially, alkaloids have attracted much attention of natural product chemists and pharmacologists due to their extensive biological activities, ranging from antifungal activities to cytotoxicity. The earlier phytochemical studies on the plants of the *Lagerstroemia indica* initiated in 1962. Ferris et al. had reported a series of phenylquinolizidine alkaloids from the aerial of title species [3–8]. Recently, several new biphenylquinolizidine alkaloids were also isolated from the aerial parts of *L. indica* by Bae et al. [9, 10]. It is interesting to note that the chemical constituents of the flowers of *L. indica* are not reported till now.

**Fig. 1:**
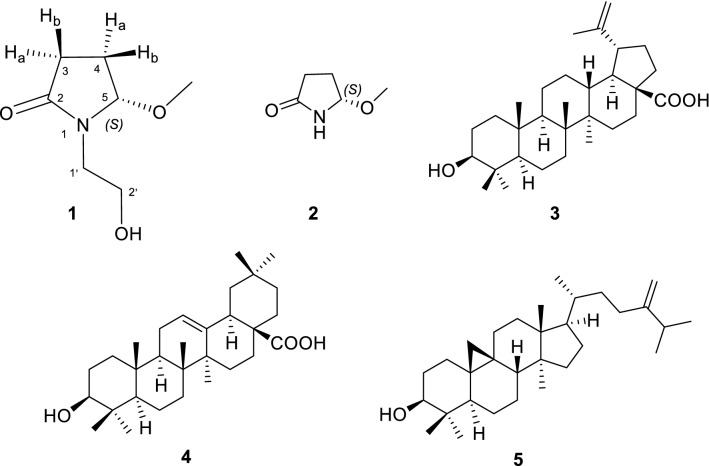
Structures of compounds **1**–**5**

In our continuous efforts towards the searching for novel bioactive secondary metabolites from marine and terrestrial sources [11–14], we have recently collected the fresh flowers of *L. indica* L. from Hunan Province, China, and carried out a chemical investigation on the EtOH extract of the flowers. As a result, a new pyrrole alkaloid, namely lagerindicine (**1**), and four known compounds (**2**–**5**) were isolated and characterized. Herein, we reported the isolation, structural elucidation, especially total synthesis of **1**, and in vitro biological evaluation of these isolates (Fig. 1).

## Results and Discussion

The *n*-BuOH and EtOAc soluble portion of the EtOH extract was repeatedly column chromatographed over silica gel, Sephadex LH-20, and RP-HPLC to yield pure compounds **1**, **2**, and **3**–**5**, respectively. The structures of the known compounds **2**–**5** were rapidly identified as pterolactam [15], betulinic acid [16], oleanolic acid [17] and 24-methylenecycloartanol [18], respectively, by the comparison of their spectral data and optical rotations with those reported in the literature.

**Fig. 2:**
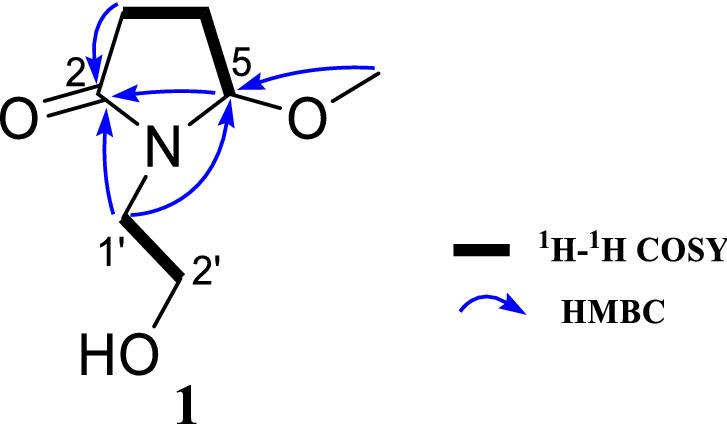
^1^H-^1^H COSY and the key HMBC correlations of compound **1**

Compound **1**, named lagerindicine, was isolated as a colorless oil. Its molecular formula was determined to be C_7_H_13_O_3_N by the ^13^C NMR data and HR-ESIMS positive ion at *m*/*z* 182.0782 [M + Na]^+^ (calcd. for C_7_H_13_NO_3_Na, 182.0788), indicating two degrees of unsaturation. Consistent with this data, analysis of the ^13^C NMR and the HSQC spectra revealed the presence of 7 carbon signals, comprising one sp^3^ methyl carbon (oxygenated at *δ*_C_ 53.7), four sp^3^ methylene carbons (one oxygenated at *δ*_C_ 60.5), one sp^3^ methine carbon (oxygenated at *δ*_C_ 92.8), and one carbonyl carbon resonating at *δ*_C_ 178.2 (Table 1). The ^1^H NMR data showed resonance of one methoxyl singlet at *δ*_H_ 3.31 (3H, s, –OMe). The four protons resonating at *δ*_H_ 3.67 (2H, each t, *J* = 5.8 Hz, H-2a’ and H-2b’), *δ*_H_ 3.55 (1H, dt, *J* = 14.0, 5.8 Hz, H-1a’), and *δ*_H_ 3.27 (1H, dt, *J* = 14.0, 5.8 Hz, H-1b’), along with the corresponding carbons at *δ*_C_ 60.5 (C-2′) and 44.3 (C-1′), indicated the presence of hydroxyethyl group. Furthermore, the four protons resonating at *δ*_H_ 2.50 (1H, dt, *J* = 17.3, 8.6 Hz, H-3a), *δ*_H_ 2.32 (1H, ddd, *J* = 17.3, 9.9, 2.8 Hz, H-3b), *δ*_H_ 2.21 (1H, dddd, *J* = 13.8, 9.9, 8.6, 6.1 Hz, H-4a), and *δ*_H_ 2.02 (1H, dddd, *J* = 13.8, 8.6, 2.8, 1.2 Hz, H-4b), together with the coupled oxymethine proton at *δ*_H_ 5.08 (1H, dd, *J* = 1.2, 6.1 Hz, H-5), suggested the direct connection from C-3 to C-5 through C-4. The above observations were further supported by the ^1^H-^1^H COSY correlations (Fig. 2). In HMBC spectrum, the correlation between –OMe to C-5 (*δ*_C_ 92.7) suggested the methoxyl locating at C-5. All the above NMR data closely resembled those of the co-occurring compound **2**, pterolactam [(*S*)-5-methoxypyrrolidin-2-one], a known alkaloid previously isolated from the aerial parts of *Chrysanthemum coronarium* L. (family Compositae) [15]. In fact, **1** differs from **2** only ascribing to the substituent at N-1. Due to the replacement of the hydrogen atom of N-1 in **2** by the hydroxyethyl group in **1**, the ^13^C NMR chemical shifts of the vicinal carbons at C-2 and C-5 of **1**, respect to those of **2**, were upfield and downfield shifted (Table 1), respectively, according to the 44 mass units differences between them. The hydroxyethyl linked at N-1 in **1** was further confirmed by the observed key HMBC correlations from H_2_-1′ to C-2 and C-5 (Fig. 2). Thus, the planar structure of compound **1** was unambiguously determined as depicted in Fig. 2. Lagerindicine is *N*-hydroxyethyl derivative of **2**.

**Table 1:** ^1^H and ^13^C NMR data of compound **1**^a^ and **2**^b^

No.	**1**		**2**	
	*δ*_H_ (mult, *J* in Hz)	*δ*_C_, type	*δ*_H_ (mult, *J* in Hz)	*δ*_C_, type
2		178.2, C		181.4, C
3a	2.50 (dt, 17.3, 8.6)	29.8, CH_2_	2.40 (dt, 16.0, 8.4)	29.4, CH_2_
3b	2.32 (ddd, 17.3, 9.9, 2.8)		2.14 (ddd, 16.0, 6.4, 2.0)	
4a	2.21 (dddd, 13.8, 9.9, 8.6, 6.1)	24.8, CH_2_	2.26 (dddd, 13.2, 6.4, 8.4, 6.4)	28.8, CH_2_
4b	2.02 (dddd, 13.8, 8.6, 2.8, 1.2)		1.97 (dddd, 13.2, 8.4, 2.0, 1.2)	
5	5.08 (dd, 1.2, 6.1)	92.8, CH	4.85 (dd, 1.2, 6.4)	88.7, CH
1a′	3.55 (dt, 14.0, 5.8)	44.3, CH_2_		
1b′	3.27 (dt, 14.0, 5.8)			
2′	3.67 (t, 5.8)	60.5, CH_2_		
–OCH_3_	3.31 (s)	53.7, CH_3_	3.26 (s)	54.8, CH_3_

^a^At 400 MHz for ^1^H and 100 MHz for ^13^C NMR experiments in CD_3_OD. *δ* (ppm) were referenced to MeOH (*δ*_H_ 3.31, *δ*_C_ 49.0), assignments made by DEPT, ^1^H–^1^H COSY, HSQC, and HMBC experiments

^b^At 400 MHz for ^1^H and 100 MHz for ^13^C NMR experiments in CD_3_OD, [15]

Since there is only one chiral center (C-5) in compound **1**, the final step to complete the full structure of **1** is to determine its absolute configuration. Since the AC of co-occurring related compound **2** is 5*S* [15], it is obvious that **1** should share the same *S* absolute configuration at C-5 based on the biogenetic consideration.

**Scheme 1:**
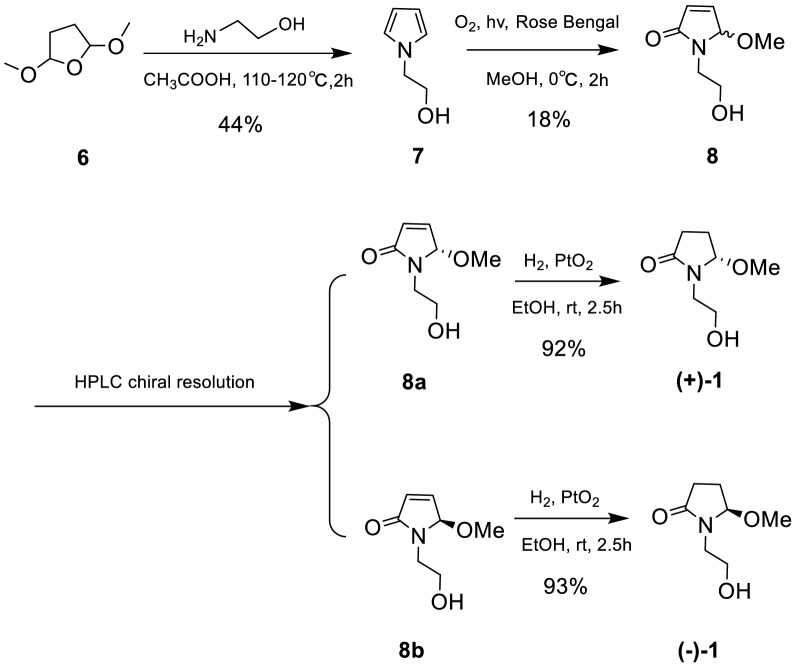
Total synthetic route of (+)-**1** and (−)-**1**

**Fig. 3:**
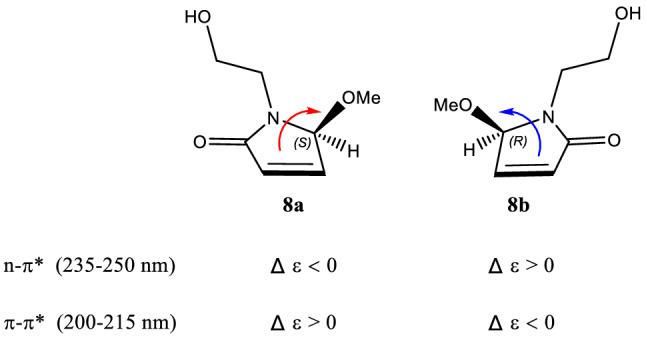
Correlation of the Cotton effects with absolute configuration

**Fig. 4:**
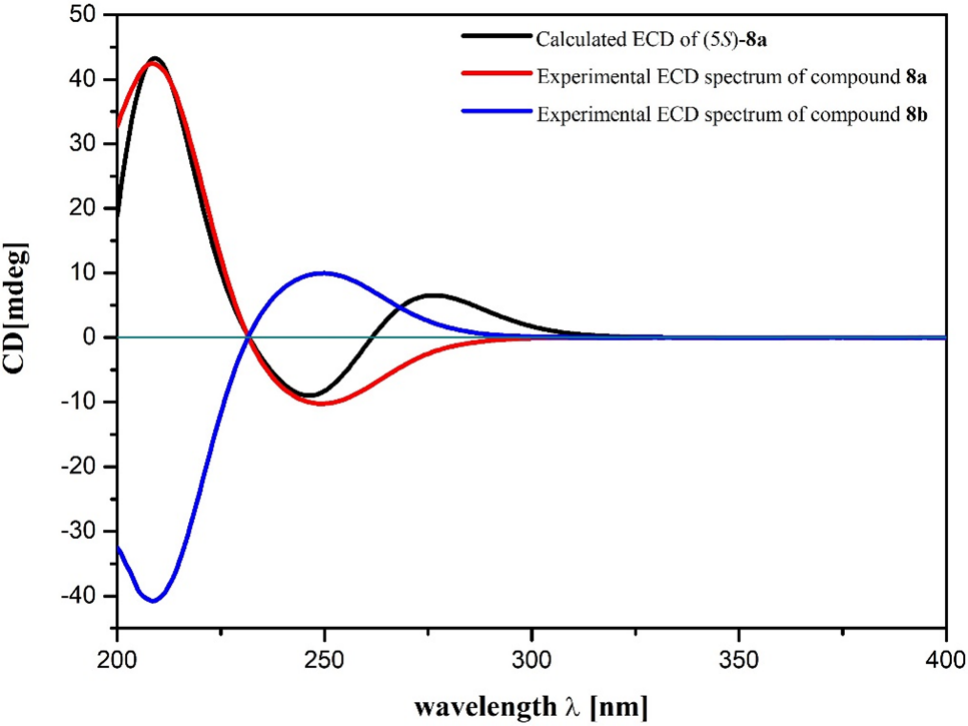
The TDDFT-ECD calculation results of **8a** and **8b**

This assignment was supported by observing the same optical rotation signs of both compounds **1** and **2** {**1**: [*α*]_D_^20^ + 13 (*c* 0.05 MeOH); **2**: [*α*]_D_^20^ + 12 (*c* 0.2 MeOH)}. However, considering the different substitution pattern at N-1, which may cause unexpected influence on the optical properties of **1** and **2**, we decided to synthesize **1** so as to confirm the deduced absolute stereochemistry of **1**. As shown in Scheme 1, a total synthesis of both (+)- and (−)-enantiomers of **1** was carried out. Thus, the key intermediate 2-pyrrol-1-ylethan-1-ol (**7**) was prepared starting from the commercially available 2,5-dimethoxytetrahydrofuran (**6**) [19]. The photooxidation of pyrrole **7** in the presence of Rose Bengal (RB, sensitizer) and methanol as polar solvent under irradiation with a 250 W long arc mercury lamp afforded (±)-5-methoxylactam (**8**) [20], which, in turn, was further separated by the chiral-phase HPLC to generate the expected (+)-5-methoxylactam {**8a**, [*α*]_D_^20^ + 131 (*c* 0.1 MeOH)} and (−)-5-methoxylactam {**8b,** [*α*]_D_^20^ − 121 (*c* 0.1 MeOH)}, respectively. Furthermore, as the presence of a *α,β*-unsaturated lactone chromophore in structures **8a** and **8b**, the absolute configuration of this pair of optically pure enantiomers could be further determined to be 5*S* and 5*R*, respectively, by the observed opposite Cotton effects due to the n–π* and π–π* transitions (Fig. 3) [21], in combination with the results of TDDFT-ECD calculation (Fig. 4), respectively. Having confirmed the AC at C-5, the subsequent reduction of the olefin of **8a** and **8b** with PtO_2_ in EtOH yielded the target molecule (+)-**1** and its enantiomer (−)-**1**, respectively. Finally, overall comparison of the physical and chemical data of synthetic (+)-**1** with those of natural product **1** confirmed the 5*S* absolute stereochemistry of natural **1**.

Considering that the promising biological activities of pyrrole alkaloids [22], we have performed in vitro biological evaluation of all the isolated compounds on human colon cancer cell line (HCT-116). The cytotoxic effects were evaluated against HCT-116 with TAK-243 (MLN7243) as the positive control. As shown in Table 2, only compound **3** showed weak cytotoxicity with the IC_50_ value of 28.4 *μ*M. Furthermore, other pharmaceutical screens, such as anti-microbial, anti-inflammatory, and insecticidal assays are undergoing.

**Table 2:** The inhibitory effect of the isolated compounds on the proliferation of HCT-116

Compounds	IC_50_ (μM)^a^
	HCT-116
**1**	> 50
**2**	> 50
**3**	28.4
**4**	> 50
**5**	> 50
TAK-243^b^	0.002

^a^The results are averages of two independent experiments

^b^TAK-243 (MLN7243) was used as a positive control

In conclusion, this is the first phytochemical investigation on the flowers of *L. indica* L. Interestingly, the previously reported metabolites from the different parts of the title plant, such as phenyl quinolizidine alkaloids, flavonoids, and flavone glycosides, etc. were not found in the present work. Moreover, it is noteworthy that pyrrole alkaloids **1** and **2** were isolated from the plants belonging to family Lythracea for the first time. The real origin of **1** is a matter worth to discuss since having used EtOH in the extraction process. To rule out the possibility that **1** may be an artificial product, we have tried to treat compound **2** by dissolving it in EtOH and stirred overnight at room temperature, but failed to detect the presence of **1**.

Further study should be conducted to extend the screening range to other bioassays such as anti-inflammatory, anti-microbe etc*.* on alkaloids **1** and **2** since the supply issue of these intriguing compounds has been solved by synthetic method.

## Experimental

### General Experimental Procedures

Optical rotations were measured on a Perkin-Elmer 241MC polarimeter (PerkinElmer, Fremont, CA, USA). ECD spectra were recorded on a Jasco J-810 spectropolarimeter (JASCO, Japan) at ambient temperature using chromatographic grade CH_3_OH and acetonitrile as solvent. IR spectra were recorded on a Nicolet Magna FT-IR 750 spectrometer (Thermo Scientific, Waltham, MA, USA); peaks are reported in cm^–1^. The NMR spectra were measured at 300 K on a Bruker DRX-400 (400 MHz for ^1^H and 100 MHz for ^13^C) spectrometer (Bruker Biospin AG, Fӓllanden, Germany), Chemical shifts (δ) are reported in parts per million (ppm) in CD_3_OD and coupling constants (*J*) in Hz, with the residual CH_3_OH (*δ*_H_ = 3.31 and *δ*_C_ = 49.0) as internal standard. LR-ESIMS and HR-ESIMS were carried out on a Bruker Daltonics Esquire 3000 plus instrument (Bruker Daltonics K. K., Kanagawa, Japan) and a Waters Q-TOF Ultima mass spectrometer (Waters, MA, USA), respectively. The semi-preparative HPLC was performed on an Agilent 1260 series liquid chromatography equipped with a DAD G1315D detector at 210 and 254 nm, and a semi-preparative ODS-HG-5 column [5 μm, 250 × 9.4 mm] was also employed. Commercial silica gel (Qingdao Haiyang Chemical Co., Ltd., Qingdao, China, 200–300 mesh, 300–400 mesh) was used for column chromatography, Sephadex LH-20 gel (Amersham Biosciences) was used for column chromatography and precoated silica gel plates (Yan Tai Zi Fu Chemical Group Co., Yantai, China, G60 F-254) were used for analytical TLC. All solvents used for extraction and isolation were of analytical grade.

### Plant Material

The flowers of *L. indica* L. were collected from the experimental forest farm of Hunan Academy of Forestry, Hunan province, China, in July 2019. The plant was identified by Dr Xiao-Ming Wang (Hunan Academy of Forestry, Hunan, China). A voucher specimen (No. 19-HN-1) was deposited at the Shanghai Institute of Materia Medica, Chinese Academy of Sciences.

### Extraction and Isolation

The fresh flowers (10 kg) were picked off on the morning, and then dried at room temperature. The dried flowers were crushed and extracted exhaustively three times with 95% EtOH (3 × 5 L), each time for 24 h. After evaporation of the solvent under vacuum, the concentrate was suspended in H_2_O (2 L), acidified to pH 4~5 with H_2_SO_4_, extracted three times with EtOAc (3 × 2 L). The EtOAc extract (59.9 g) was obtained after removing the solvents under vacuum. The aqueous layer was then basified with Na_2_CO_3_ to pH 9~10 and extracted three times with CHCl_3_ (3 × 2 L), after filtration, the organic solvents were removed under vacuum to give the CHCl_3_ extract (0.5 g). The aqueous layer was further extracted three times with *n*-BuOH (3 × 2 L). After filtration, the organic solvents were removed under vacuum to give the *n*-BuOH extract (5.0 g). The *n*-BuOH extract was successively separated by silica column chromatography (CC) (column: 30 × 6 cm, eluting with petroleum ether: Acetone = 100:0 → 0:100), Sephadex LH-20 CC (column: 150 × 4 cm, eluting with dichloromethane: methanol = 1:1), and reversed-phase HPLC (CH_3_CN:H_2_O = 5:95) to afford compound **1** (3.6 mg, *t*_R_ = 16.0 min) and compound **2** (4.1 mg, *t*_R_ = 14.6 min). The EtOAc extract was repeatedly separated via silica CC (column: 30 × 6 cm, eluting with petroleum ether: diethyl ether = 100:0 → 0:100), and Sephadex LH-20 CC (column: 150 × 4 cm, eluting with petroleum ether: dichloromethane: methanol = 2:1:1) to obtain compound **3** (4.8 mg), compound **4** (38.0 mg) and compound **5** (10.3 mg).

#### Lagerindicine (1)

Colorless oil; [*α*]_D_^20^ + 13 (*c* 0.05 MeOH); UV (MeOH) *λ*_max_ (log *ε*) 210 (3.10) nm, *λ*_max_ (log *ε*) 270 (2.20) nm; IR: *v* = 3418, 2932, 1682, 1456, 1373, 1259, 1175, 1076, 800 cm^−1^; ^1^H and ^13^C NMR data see Table 1; HR-ESIMS *m/z* 182.0782 [M+ Na]^+^ (calcd. for C_7_H_13_NO_3_Na, 182.0788).

#### Pterolactam (2)

White amorphous powder (MeOH); [*α*]_D_^20^ + 12 (*c* 0.2 MeOH); Lit [15]: [*α*]_D_^20^ + 10 (*c* 0.1, MeOH). ^1^H NMR and ^13^C NMR data see Table 1.

### Synthesis Procedures

#### 2-Pyrrol-1-ylethan-1-ol (**7**)

Ethanolamine (6.6 mL, 0.1 mmol) was added to glacial acetic acid (12 mL) at 0 °C, and kept the temperature at 15–25 °C. Then, 2,5- dimethoxytetrahydrofuran (**6**) (3.4 mL, 0.02 mmol) was added and the reaction was stirred at 110 − 120 °C. After 2 h at that temperature, the residual liquid in the reaction vessel was cooled to rt and water was added. The usual work-up (DCM, brine, saturated aqueous Na_2_CO_3_) afforded a residue that was dissolved in MeOH (6.7 mL). NaOH (20 wt %, 3.3 mL) was added, and the solution was stirred at rt for 1 h, then poured into brine, and extracted with DCM. The solvent was removed under reduced pressure to afford **7** (1.2 g, 44%) as a yellow oil. The spectroscopic data of **7** are as follows: ^1^H NMR (400 MHz, CDCl_3_) *δ*_H_ 6.70 (t, 2H), 6.18 (t, 2H), 4.00 (t, 2H), 3.81 (t, 2H) ppm.

#### 1-(2-Hydroxyethyl)-5-methoxy-1*H*-pyrrol-2(5*H*)-one (**8**)

A solution of the compound **7** (1.8 mmol) in methanol (50 mL) containing a catalytic amount of sensitizer Rose Bengal (RB) was placed in a flask, and oxygen was gently bubbled through it. The solution was cooled to 0 ℃ and irradiated with a 250 W long arc mercury lamp for 2 h. The photooxidations gave complex mixtures of oxygenated products, and compound **8** (49.9 mg, 18%) were purified by flash column chromatography using silica gel (petroleum ether/acetone = 5:1 → 1:1 v/v). **8a** {20.1 mg, [*α*]_D_^20^ + 131 (*c* 0.1 MeOH), *t*_R_ = 7.5 min} and **8b** {20.3 mg, [*α*]_D_^20^ − 121 (*c* 0.1 MeOH), *t*_R_ = 10.0 min} were obtained through the chiral-phase HPLC resolution of **8** (CHIRAPAK IC, Lot No. IC00CE-QH040) (20% isopropanol, flow rate: 2 mL/min). The spectroscopic data of **8** are as follows: ^1^H NMR (400 MHz, CDCl_3_) *δ*_H_ 6.90 (dd, 1H, *J* = 1.6, 6.1 Hz), 6.23 (dd, 1H, *J* = 0.7, 6.1 Hz), 5.47 (br s, 1H), 3.73 (m, 2H), 3.56 (m, 1H), 3.41 (m, 1H), 3.12 (s, 3H) ppm; ^13^C NMR (100 MHz, CDCl_3_) *δ*_C_ 170.7, 144.1, 130.3, 89.3, 61.3, 51.0, 43.1 ppm

#### (±)-Lagerindicine (**1**)

Compound **8a** (9.6 mg, 0.067 mmol) and **8b** (11.5 mg, 0.071 mmol) in 5 mL of EtOH were reduced with H_2_ at room temperature, using the catalytic amount of PtO_2_ (2.0 mg) as catalyst, respectively. At the end of 2.5 h, the mixtures were filtered. Upon removed of the EtOH, compounds (+)-**1** {8.9 mg, [*α*]_D_^20^ + 6.8 (*c* 0.1 MeOH), 92%} and (−)-**1** {10.9 mg, [*α*]_D_^20^ −12.7 (*c* 0.1 MeOH), 93%) were obtained, respectively. The NMR spectra of (+)-**1** and (−)-**1** see Figure S19–22.

### Cytotoxicity Assays

The cytotoxicity of the isolated compounds was evaluated against the HCT-116 by using the sulforhodamine B (SRB) method, according to the protocols described in previous literature [23]. The cytotoxicity of the compounds was expressed as the half-maximal inhibition (IC_50_, μM). TAK-243 (MLN7243) was used as a positive control.

## Electronic supplementary material

Below is the link to the electronic supplementary material.

Supplementary file1 (DOCX 1245 kb)
