# Supplementary material for: Lagerindicine, a New Pyrrole Alkaloid Isolated from the Flowers of Lagerstroemia indica Linnaeus
Source: Nat Prod Bioprospect. 2020 Oct 20;11(1):73–9. doi: 10.1007/s13659-020-00273-x (PMC7933331; doi:10.1007/s13659-020-00273-x)
Supplement: Supplementary file 1 — Supplementary file1 (DOCX 1245 kb) [file 13659_2020_273_MOESM1_ESM.docx]

**Supplementary material**

**Lagerindicine, a new pyrrole alkaloid isolated from the flowers of *Lagerstroemia indica* Linnaeus**

Yi Chen^b,c^, Song-Wei Li^a^, Fang-Zhou Yin^a^, Min Yang^a^, Xia-Juan Huan^a^, Ze-Hong Miao^a^, Xiao-Ming Wang^b,c*^, Yue-Wei Guo^a,d*^

^a^ State Key Laboratory of Drug Research, Shanghai Institute of Materia Medica, Chinese Academy of Sciences, 555 Zu Chong Zhi Road, Zhangjiang Hi-Tech Park, Shanghai 201203, China

*^b^* *Hunan Academy of Forestry,* *658 South shao shan Road, Changsha, Hunan 410004, China*

^c^ Changsha Engineering Technology Research Center of Woody flower, 658 South shao shan Road, Changsha, Hunan 410004, China

*^d^* *College of Materials Science and Engineering, Central South University of Forestry and Technology, 498 South Shao shan Road, Changsha, Hunan 410004, China*

**Table of contents**

[1. Chiral-phase HPLC resolution 3](#_Toc47558123)

[2. Original spectra of compound **1**. 4](#_Toc47558124)

[3. Original spectra of compound **2**. 8](#_Toc47558125)

[4. Original spectra of compounds **7** and **8**. 9](#_Toc47558126)

[5. Original spectra of compounds **(+)-1** and **(-)-1**. 11](#_Toc47558127)

[6. Computational Section. 14](#_Toc47558128)

## Chiral-phase HPLC resolution


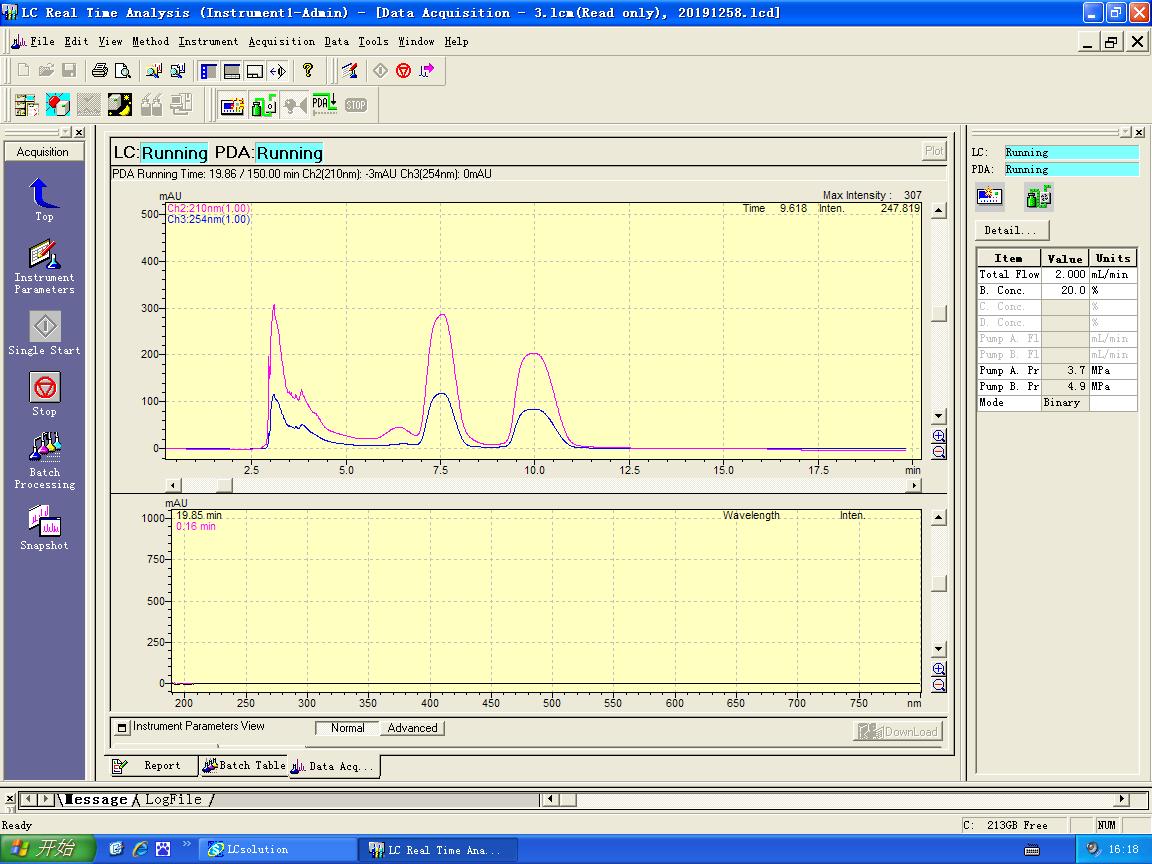


**Figure S1.** HPLC chiral resolution of **8a** (t_R_ = 7.5 min) and **8b** (t_R_ = 10.0 min).


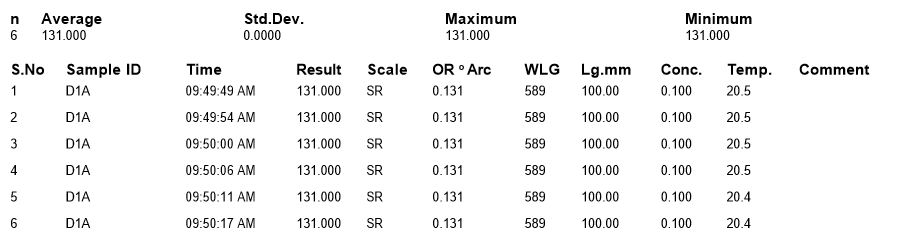


**Figure S2.** Optical rotation of **8a.**


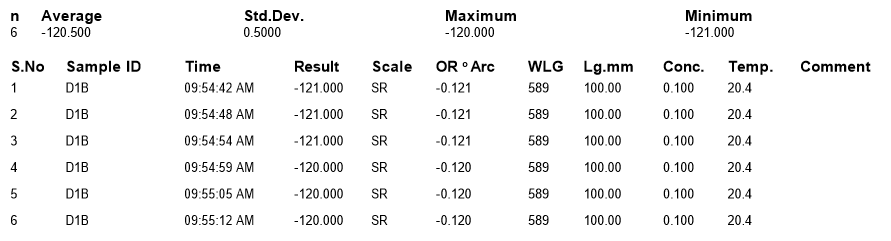


**Figure S2.** Optical rotation of **8b.**

## Original spectra of compound 1.


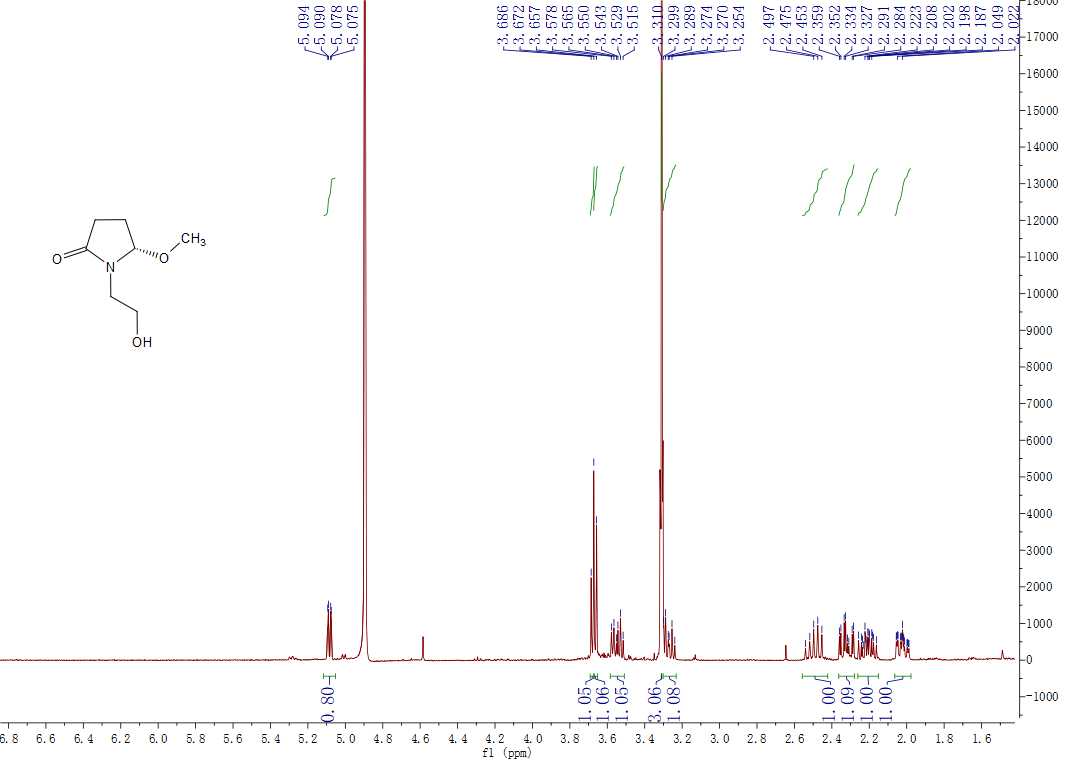


**Figure S3.** ^1^H NMR spectrum (400 MHz, CD_3_OD) of compound **1**.


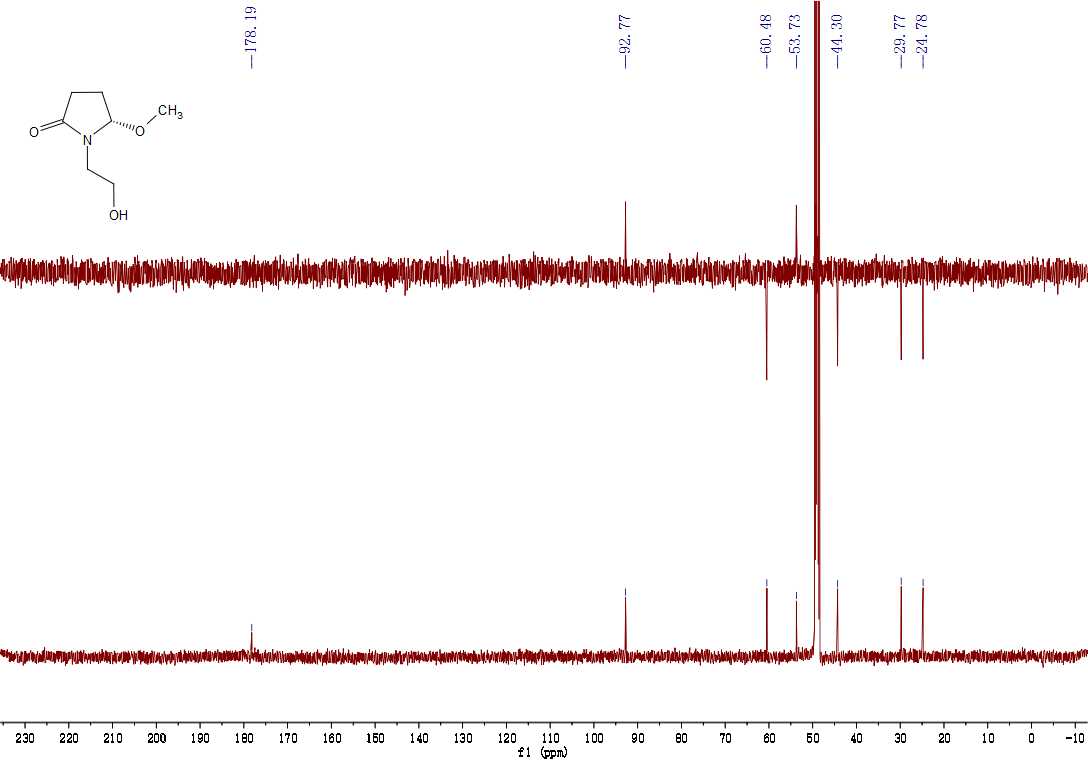


**Figure S4.** ^13^C NMR spectrum (100 MHz, CD_3_OD) of compound **1**.


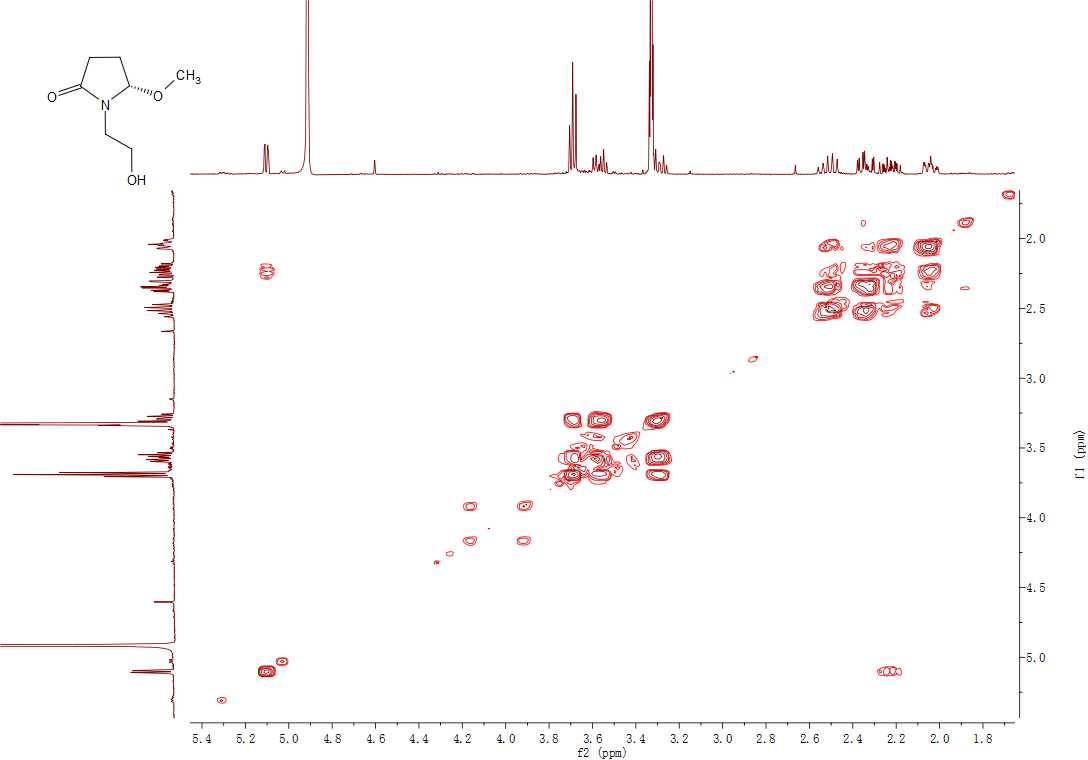


**Figure S5.** ^1^H-^1^H COSY spectrum (400 MHz, CD_3_OD) of compound **1**.


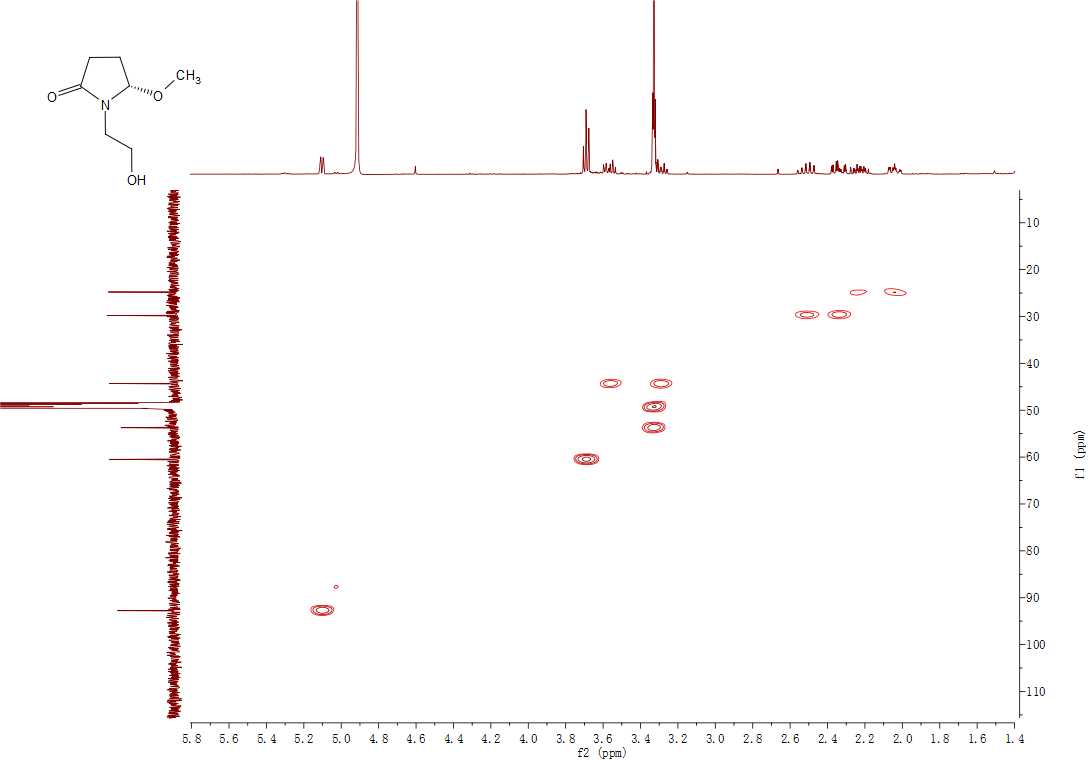


**Figure S6.** HSQC spectrum (400 MHz, CD_3_OD) of compound **1**.


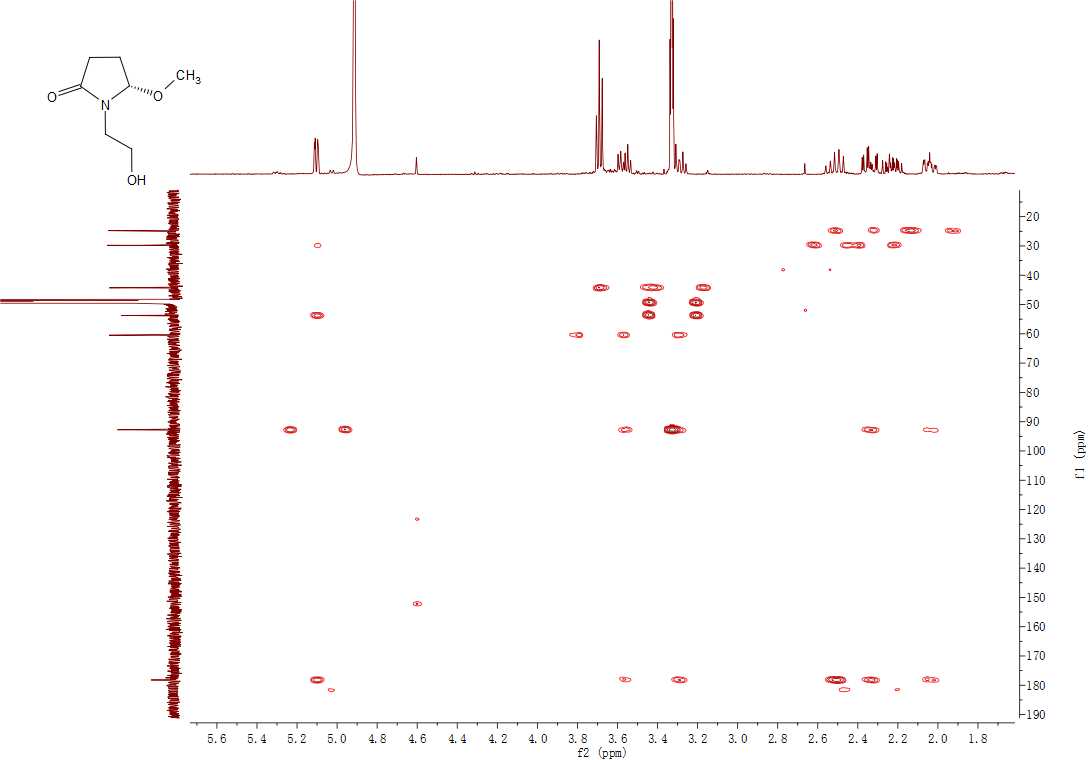


**Figure S7.** HMBC spectrum (400 MHz, CD_3_OD) of compound **1**.


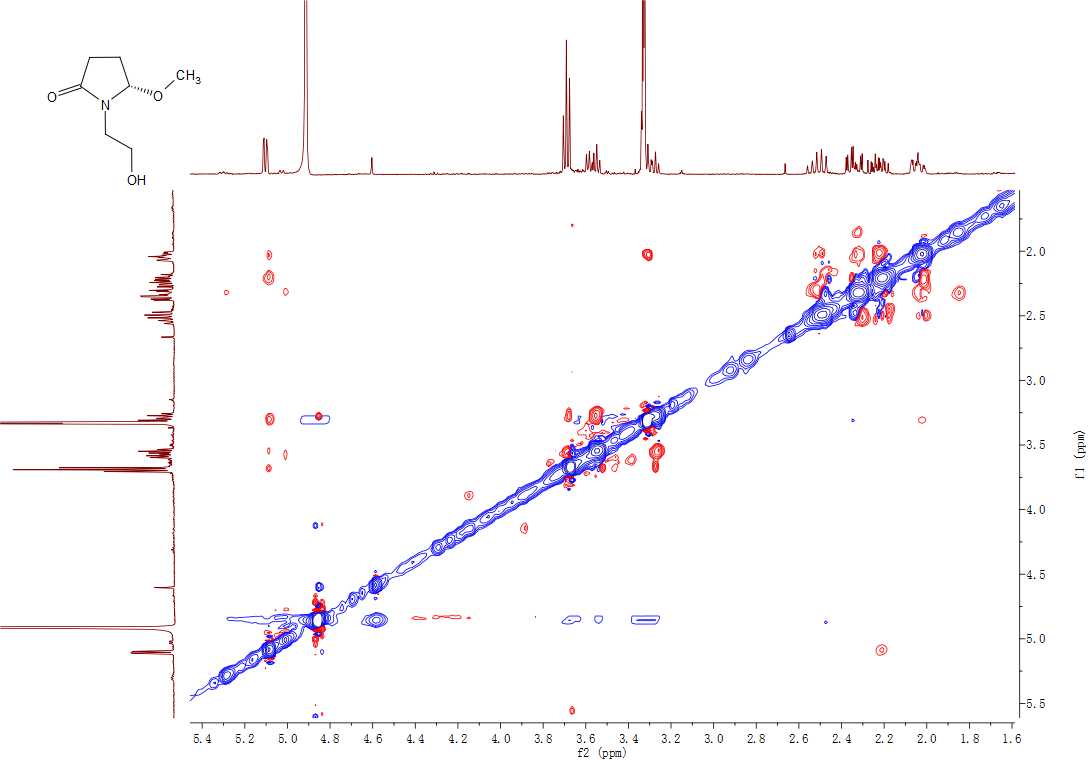


**Figure S8.** NOESY spectrum (400 MHz, CD_3_OD) of compound **1**.


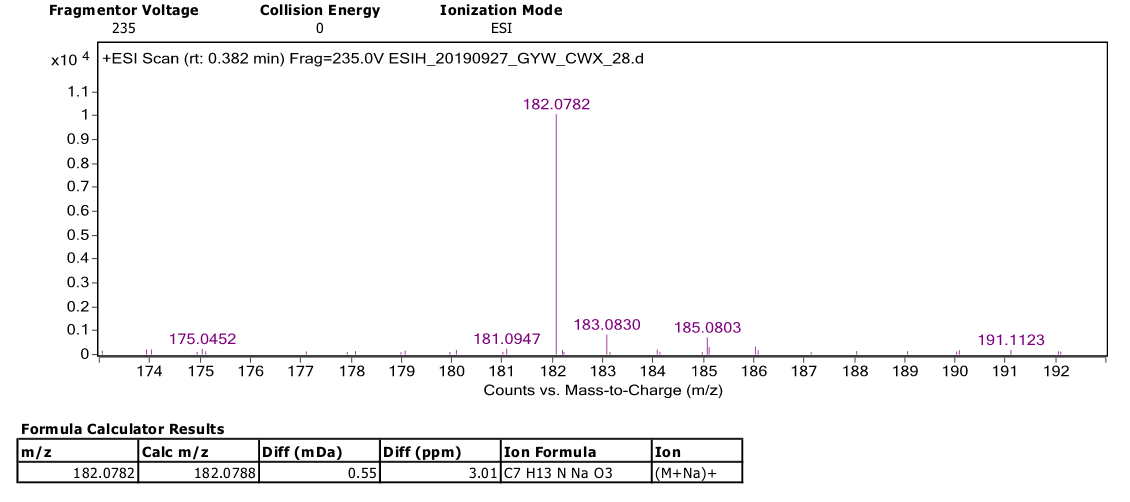


**Figure S9.** HR-ESI-MS spectrum of compound **1.**


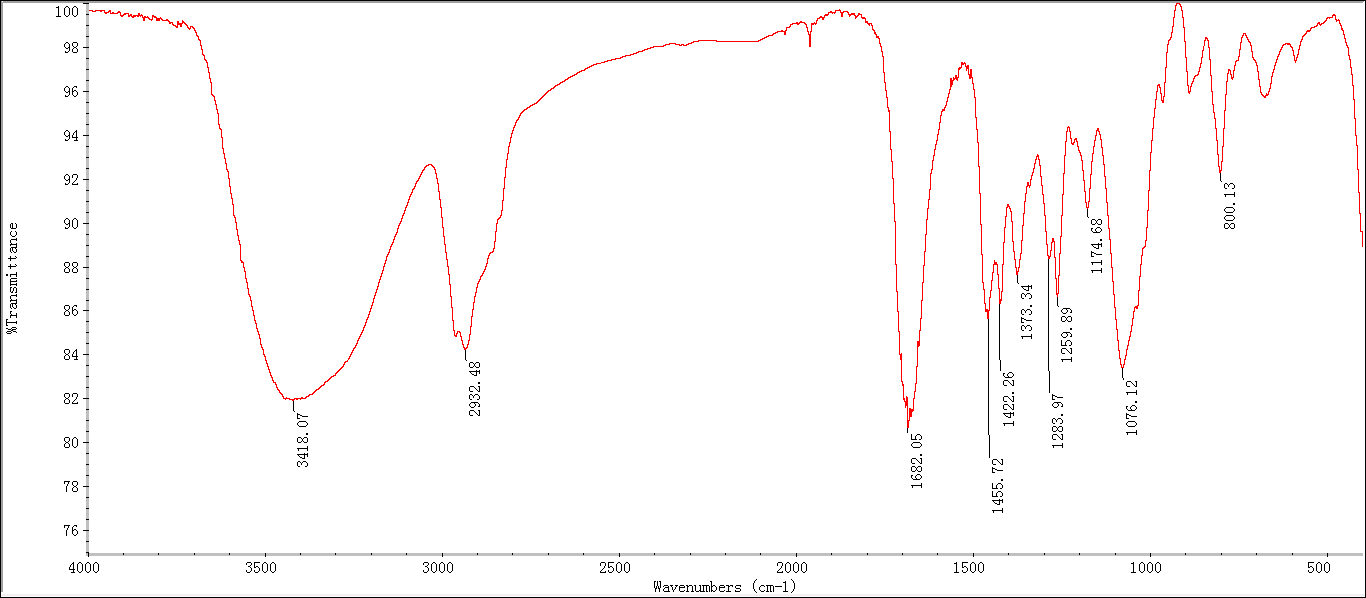


**Figure S10.** IR spectrum of compound **1.**

**
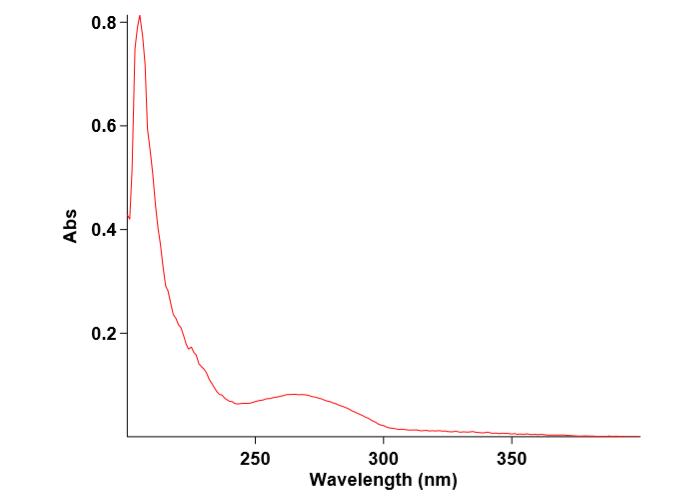
**

**Figure S11.** UV spectrum of compound **1.**

## Original spectra of compound 2.


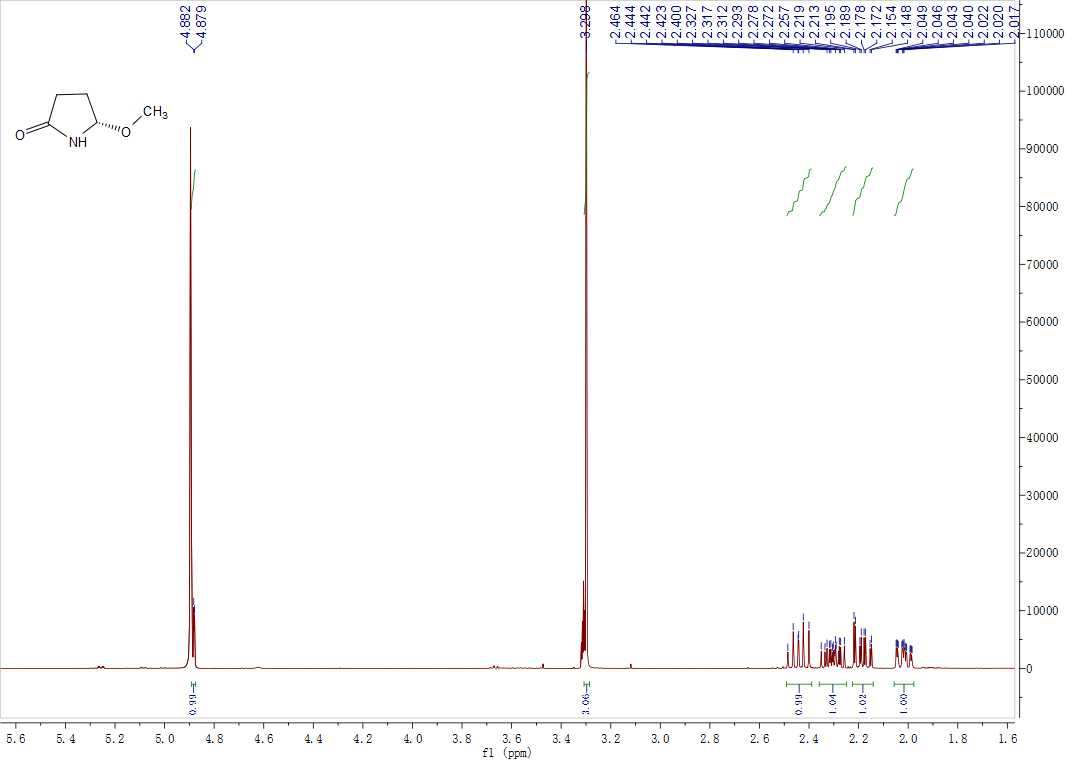


**Figure S12.** ^1^H NMR spectrum (400 MHz, CD_3_OD) of compound **2**.


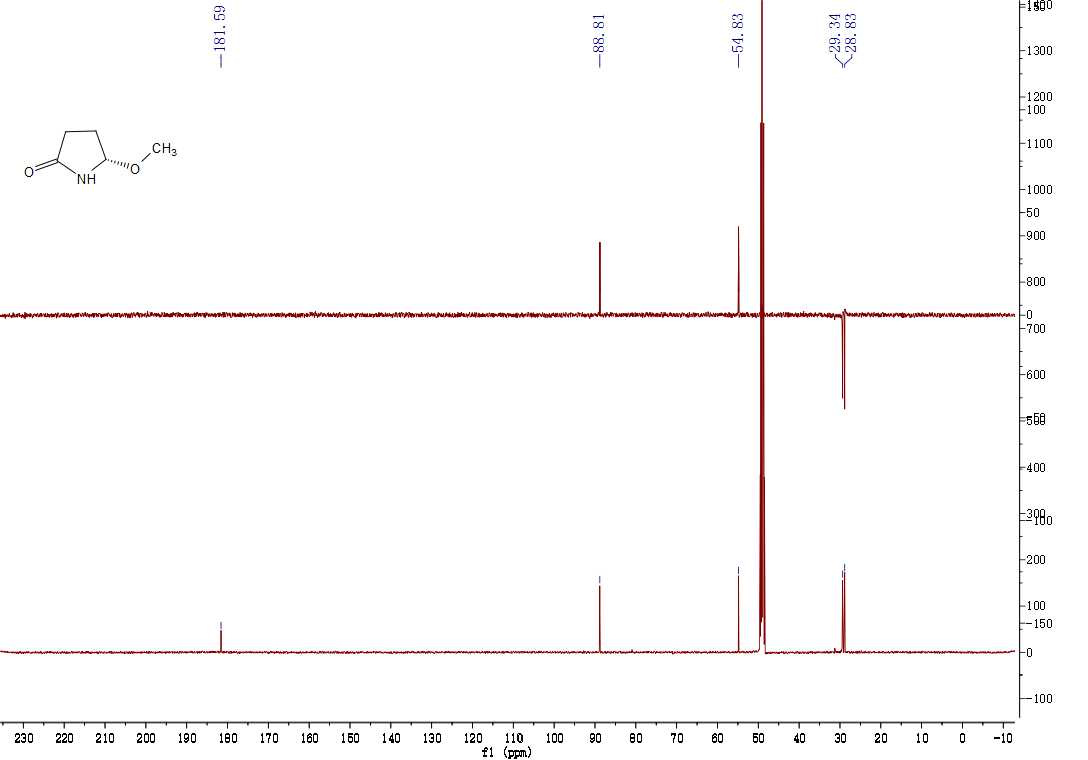


**Figure S13.** ^13^C NMR spectrum (100 MHz, CD_3_OD) of compound **2**.

## Original spectra of compounds 7 and 8.


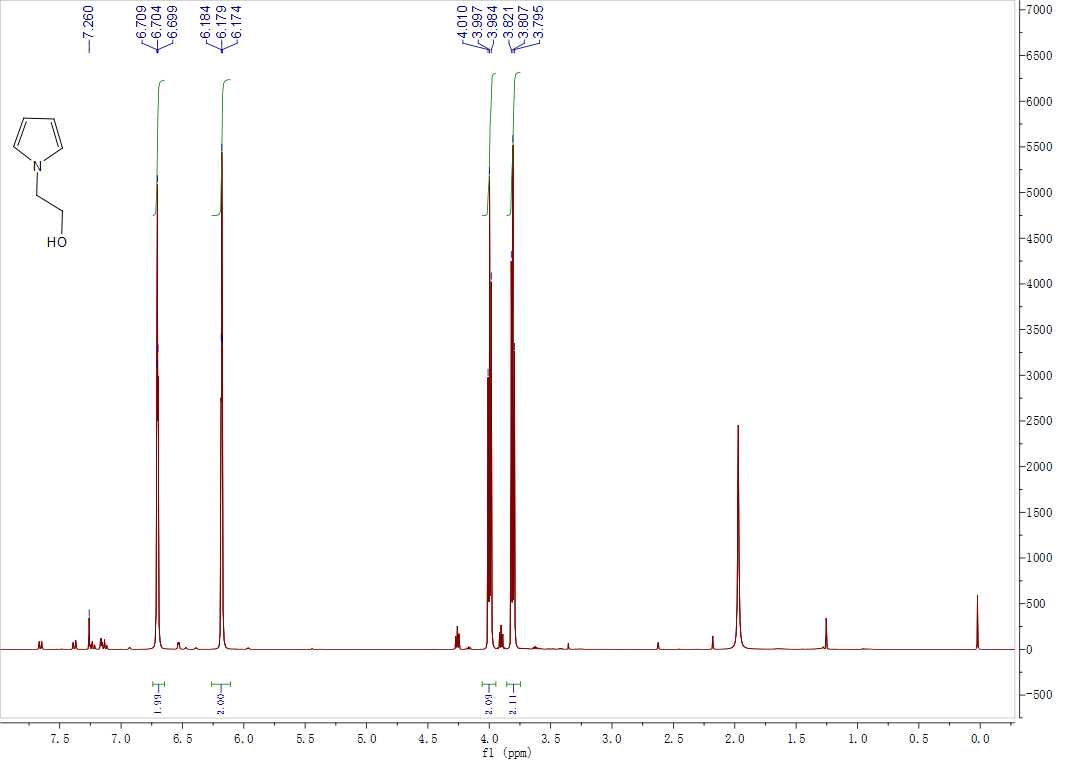


**Figure S14.** ^1^H NMR spectrum (400 MHz, CDCl_3_) of compound **7**.


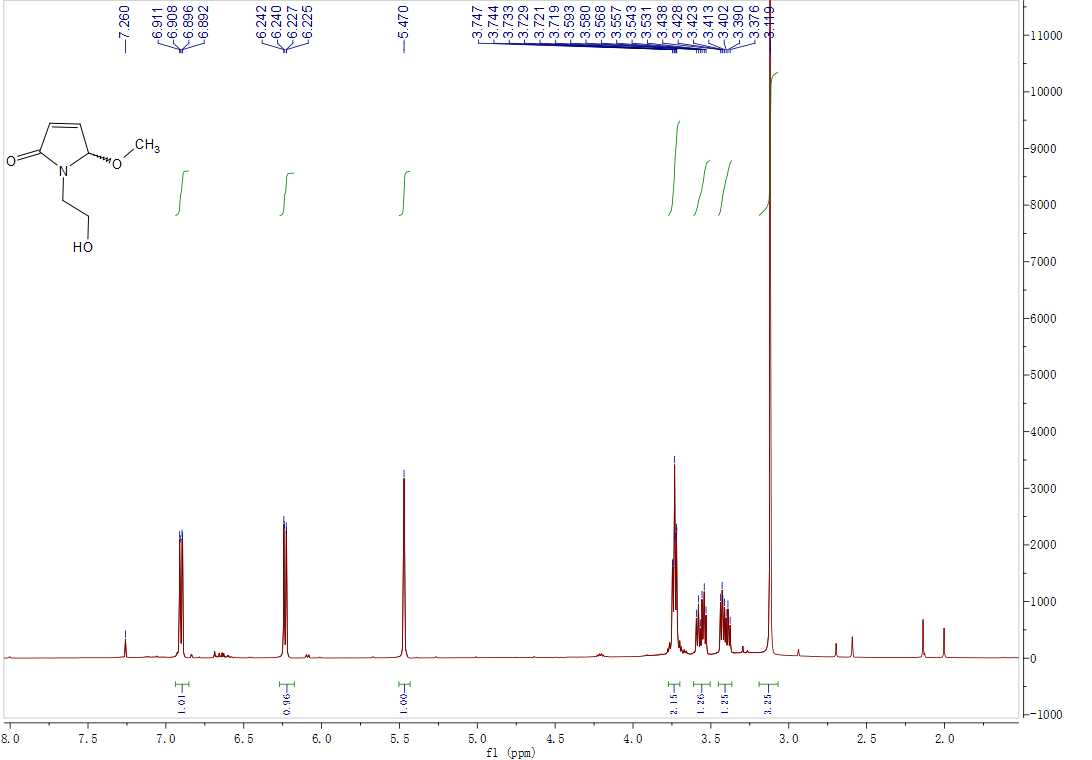


**Figure S15.** ^1^H NMR spectrum (400 MHz, CDCl_3_) of compound **8**.


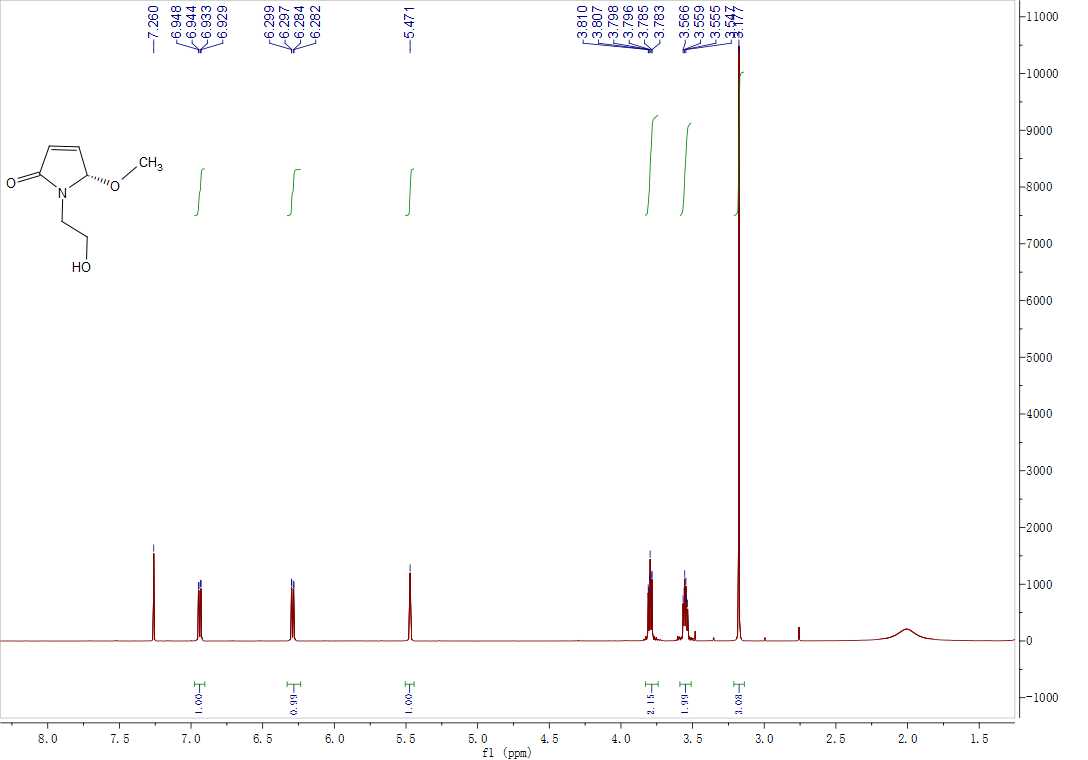


**Figure S16.** ^1^H NMR spectrum (400 MHz, CDCl_3_) of compound **8a/8b**.


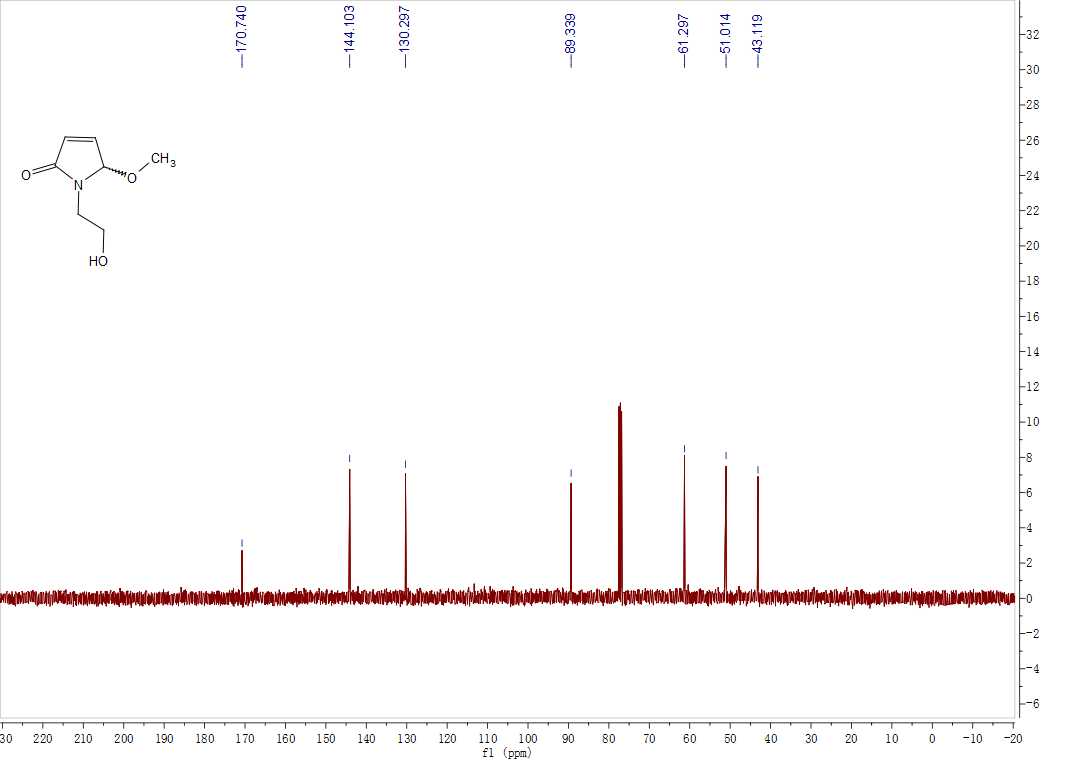


**Figure S17.** ^13^C NMR spectrum (100 MHz, CDCl_3_) of compound **8**.


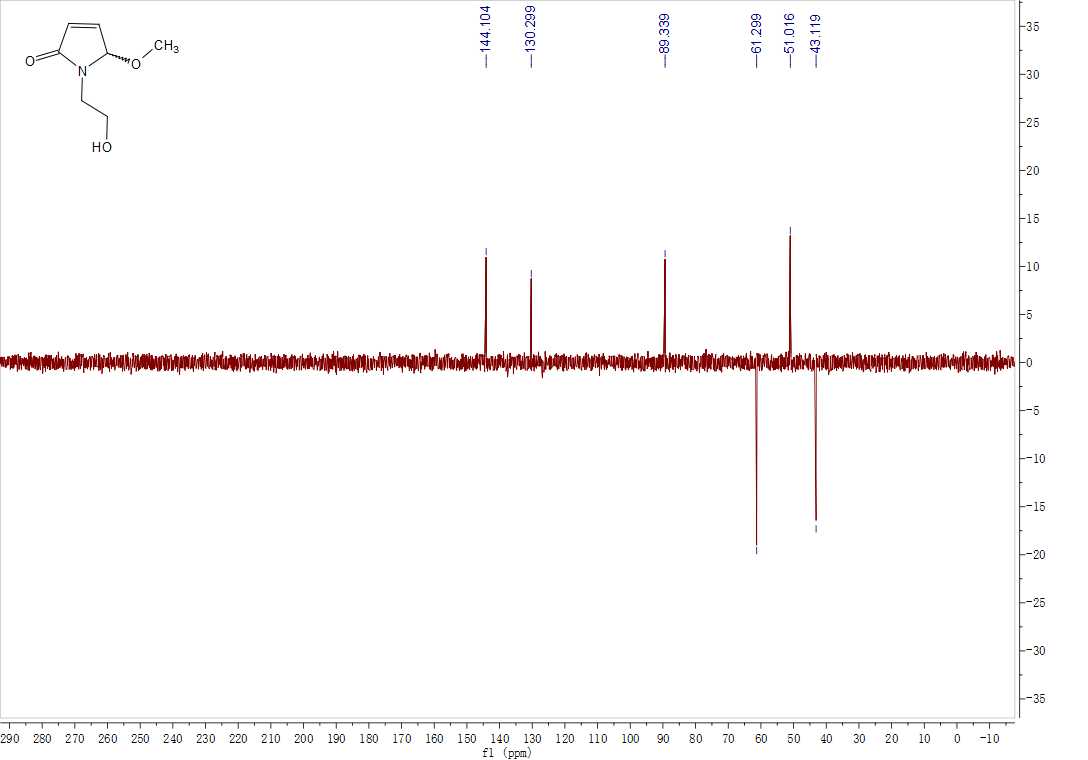


**Figure S18.** DEPT-135 NMR spectrum (100 MHz, CDCl_3_) of compound **8**.

## Original spectra of compounds (+)-1 and (-)-1.


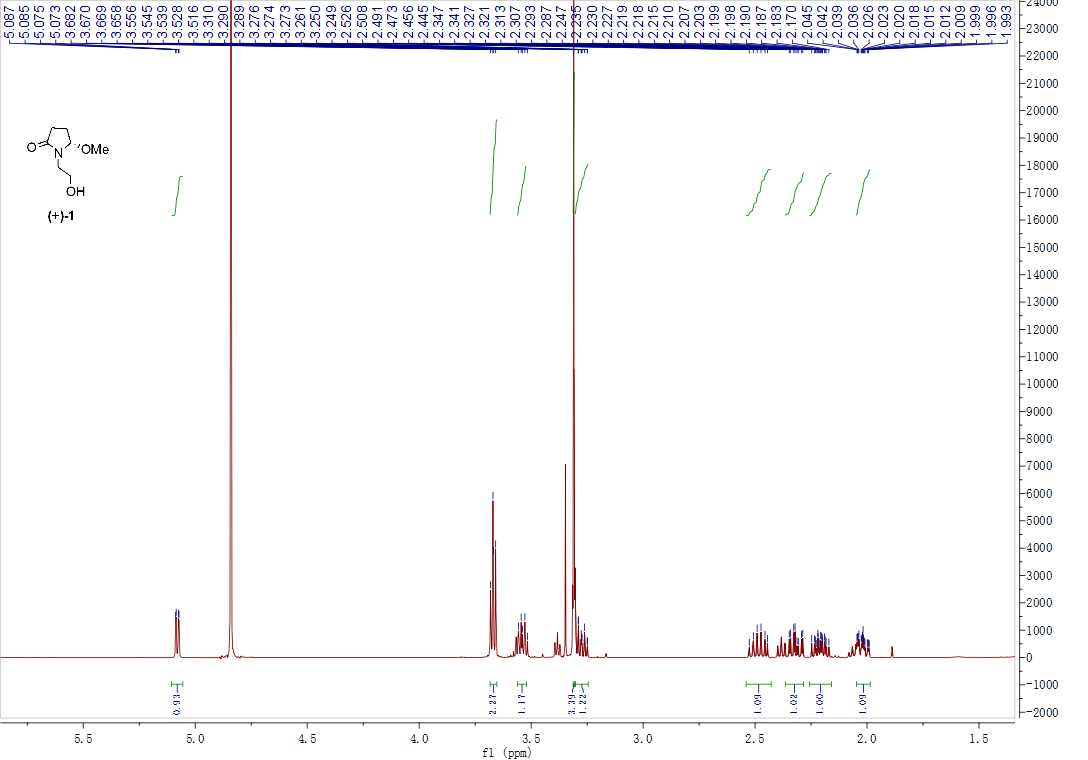


**Figure S19.** ^1^H NMR spectrum (500 MHz, CD_3_OD) of compound **(+)-1**.


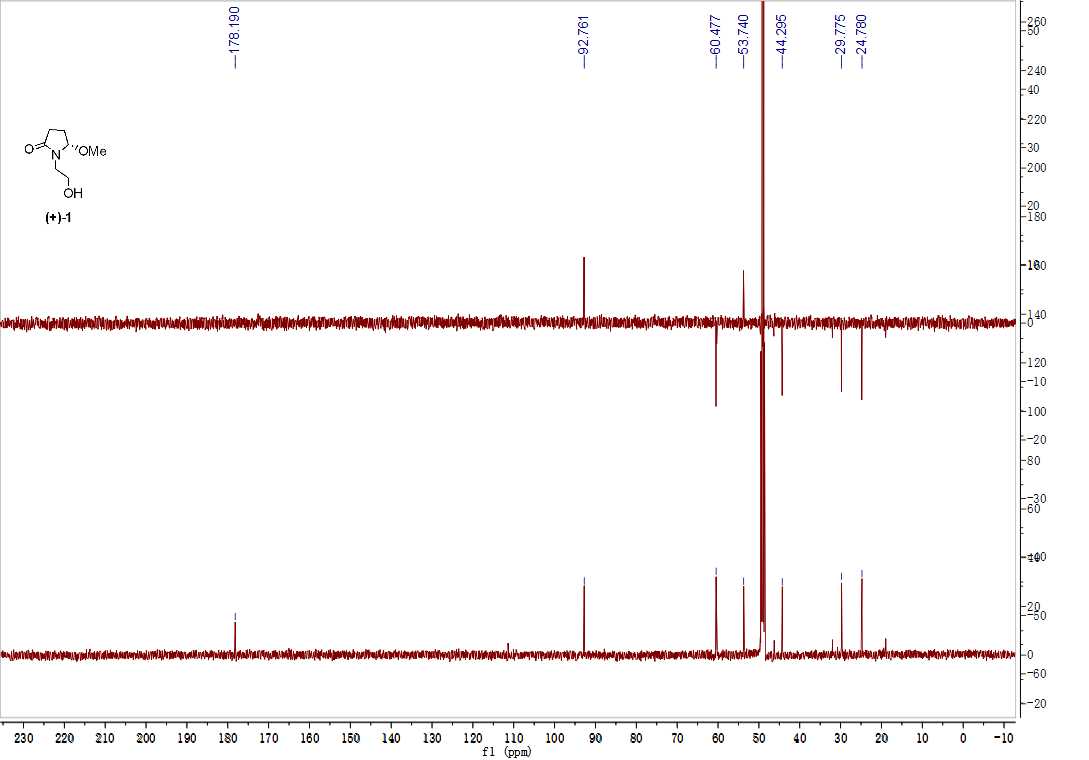


**Figure S20.** ^13^C NMR spectrum (125 MHz, CD_3_OD) of compound **(+)-1**.


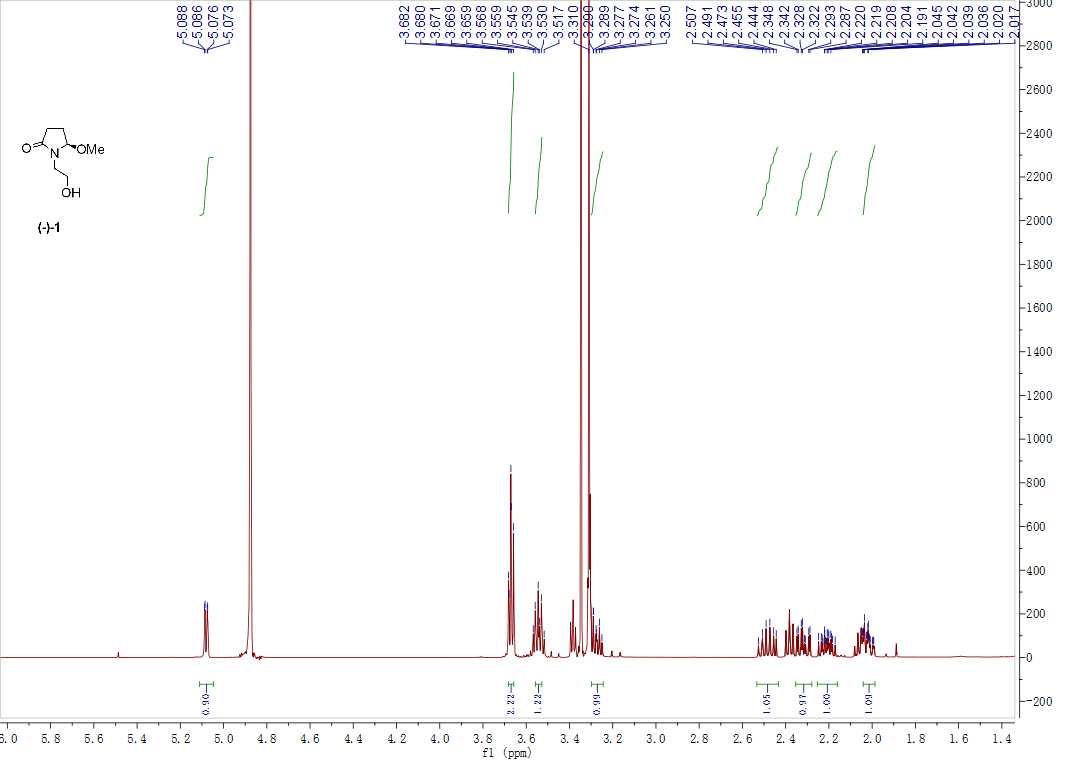


**Figure S21.** ^1^H NMR spectrum (500 MHz, CD_3_OD) of compound **(-)-1**.


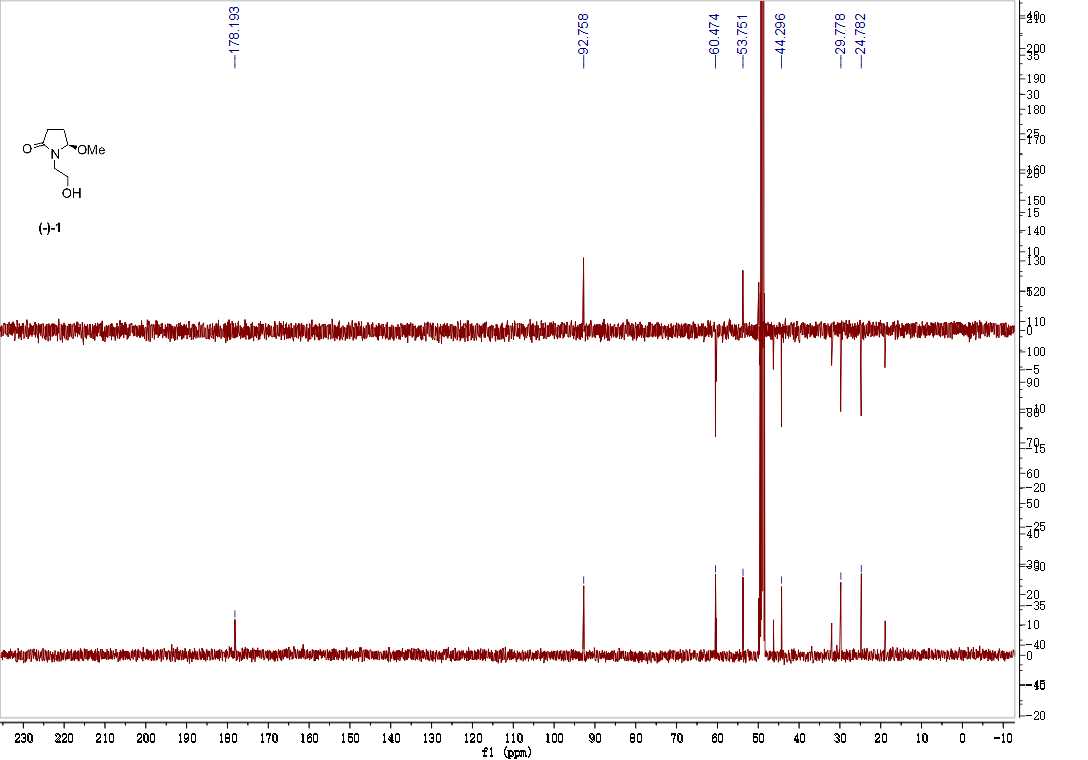


**Figure S22.** ^13^C NMR spectrum (125 MHz, CD_3_OD) of compound **(-)-1**.


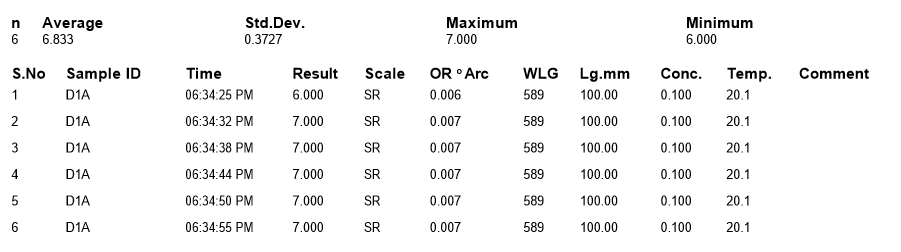


**Figure S23.** Optical rotation of **(+)-1.**


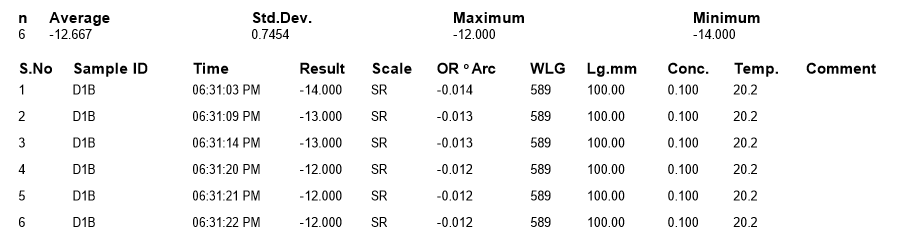


**Figure S24.** Optical rotation of **(-)-1.**

## Computational Section.


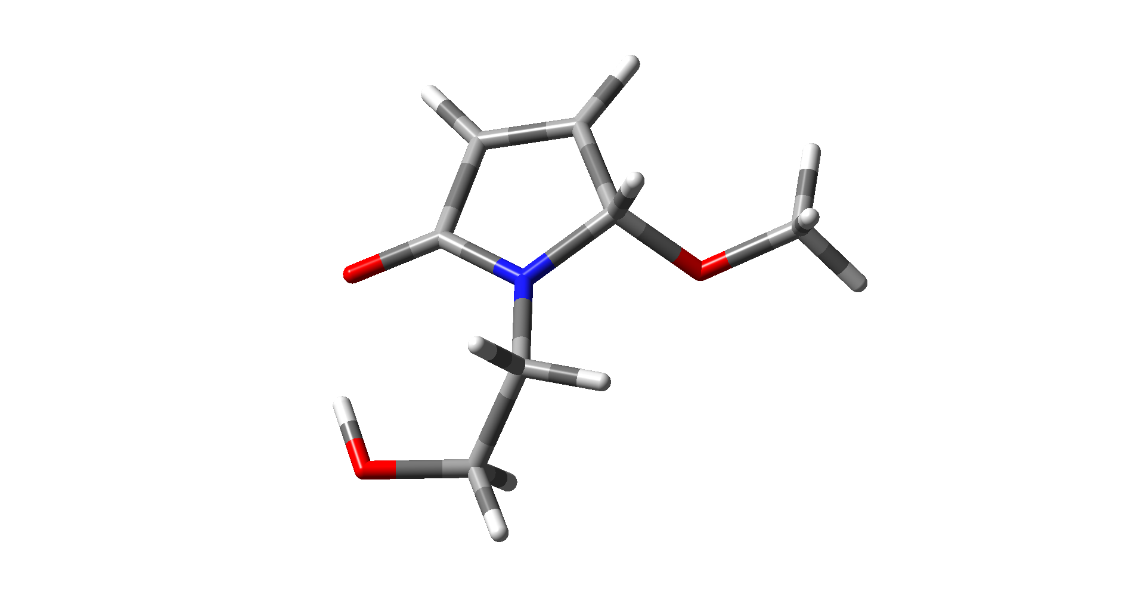


**Figure S25.** The lowest-energy conformer of (5*S*)-**8a** for TDDFT-ECD calculation.


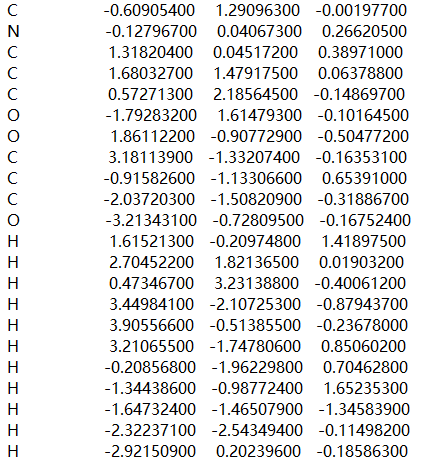


**Figure S26.** Cartesian coordinates of the lowest-energy conformer of (5*S*)-**8a**.
